# Validation of a new non-invasive predictive score (KASAI) for primary aldosteronism subtyping

**DOI:** 10.1530/EC-25-0156

**Published:** 2025-09-03

**Authors:** Guillaume Pierman, Karin Zibar Tomsic, Darko Kastelan, Carlien De Herdt, Natacha Driessens, Laurent Vroonen, Audrey Loumaye, Dominique Maiter, Raluca Maria Furnica

**Affiliations:** ^1^ Department of Endocrinology, Université Catholique de Louvain, Cliniques Universitaires St-Luc, Bruxelles, Belgium; ^2^ Department of Endocrinology, Centre Hospitalier Universitaire UCL Namur, Yvoir, Belgium; ^3^ Department of Endocrinology, University Hospital Center Zagreb, Zagreb, Croatia; ^4^ School of Medicine, University of Zagreb, Zagreb, Croatia; ^5^ Department of Endocrinology-Diabetology-Metabolism, Universiteit Ziekenhuis Antwerpen, Edegem, Belgium; ^6^ Department of Endocrinology, Université Libre de Bruxelles, Hôpital Universitaire de Bruxelles, Bruxelles, Belgium; ^7^ Department of Endocrinology, Centre Hospitalier Universitaire de Liège, Liège, Belgium

**Keywords:** primary aldosteronism subtyping, adrenal venous sampling, predictive clinical score, arterial hypertension

## Abstract

**Introduction:**

Adrenal venous sampling (AVS) is considered the gold standard test for primary aldosteronism (PA) subtyping. Considering the limited availability of this challenging procedure, we propose a noninvasive score predicting unilateral (UPA) or bilateral (BPA) form of PA in order to reduce the need for AVS.

**Material and methods:**

The score was retrospectively developed from a cohort of 72 patients who underwent AVS (21 patients with BPA and 51 with UPA) at Cliniques Universitaires Saint Luc between 1993 and 2021. Another multicenter cohort of 130 patients who underwent AVS (67 patients with BPA and 63 with UPA) served as external validation.

**Results:**

Four predictive parameters of UPA highlighted by logistic regression analysis were integrated into the KASAI score: minimal serum potassium value, supine resting aldosteronemia, aldosteronemia at the end of the saline infusion test, and results of adrenal imaging. Depending on the results, 0, 1, or 3 points were assigned to each parameter. In both cohorts, a score greater than 9/12 identified UPA and a score less than 4/12 identified BPA with 100% specificity, while performing AVS remained indicated for scores between 4 and 9. The score may have avoided AVS in 40% of patients in the primary cohort and in 42% of patients in the validation cohort. The area under the ROC curve for discrimination of UPA from BPA was 0.81 (95% CI, 0.70–0.90) in the primary cohort and 0.86 (95% CI, 0.80–0.90) in the validation cohort.

**Conclusion:**

We propose a new biological-radiological score that could simplify the diagnostic assessment of PA.

## Introduction

Primary aldosteronism (PA) is a common cause of secondary hypertension with a variable prevalence ranging from 11 to 22% in cases of resistant hypertension (1) to 88% in cases of hypertension and spontaneous hypokalemia <2.5 mmol/L (2). PA is associated with an increased cardiovascular risk (stroke, coronary artery disease, atrial fibrillation, and heart failure) compared with patients affected by essential hypertension of similar severity (2), due to the deleterious effects of aldosterone on cardiac remodeling and vascular fibrosis (3).

Despite increased awareness of the high prevalence and serious complications of PA, detection rate and appropriate treatment remain suboptimal, even in high-risk populations (4). This is partly due to the complexity of the diagnostic process, first involving the adequate assessment of an aldosterone-to-renin ratio (ARR), followed by confirmatory testing to demonstrate autonomous aldosterone secretion (5). Furthermore, it is essential to determine whether the production of aldosterone is unilateral (unilateral primary aldosteronism: UPA) or bilateral (bilateral primary aldosteronism: BPA) in order to recommend the most appropriate treatment. This specific subtype diagnosis currently relies on adrenal venous sampling (AVS), an invasive procedure associated with several challenges, including the limited availability of specialized centers, technical difficulties resulting in a highly variable success rate, the necessity to adjust antihypertensive medications, and the complexity of results interpretation due to the lack of universally accepted standardized protocols (3, 6). Alternative methods to AVS have been considered in recent years, including functional imaging, radiomics, and steroid metabolomic profiling (7). However, most of these methods are costly and primarily limited to academic settings. Thus, a recent meta-analysis evaluated the diagnostic performance of various prediction scores based on clinical, biochemical, and/or radiological parameters for identifying BPA (8) and concluded that their sensitivity was very low, limiting their use in daily practice. Furthermore, only a few of these algorithms have been tested in different cohorts, which typically lead to lower diagnostic performance (9).

In this context, we propose a new score (KASAI) able to accurately predict UPA or BPA in a significant subset of patients, therefore reducing the number of necessary AVS. We validate our predictive diagnostic score across a multicenter cohort. We also compare our score against a previously described score-based algorithm (SPACE score).

## Materials and methods

### Patient selection and data collection

Our development cohort included 72 patients over the age of 18 years (67 with age over 35) who underwent AVS for the evaluation of PA in our tertiary center between 1993 and 2021. PA was diagnosed according to the guidelines recommended at the time of evaluation, the last used being the Endocrine Society guidelines published in 2016 (5). To confirm the diagnosis, patients underwent a suppression test, which was a saline infusion test (SIT, 2,000 mL of saline IV over 4 h in a supine position) in 52 patients and a captopril test in 20 patients. A plasma aldosterone concentration (PAC) >10 ng/dL (277.4 pmol/L) at the end of the test confirmed the diagnosis. All measurements of aldosterone and renin concentrations as well as the SIT were performed in the morning, in a supine position, after withdrawal of drugs affecting the ARR (more specifically spironolactone and estrogen-containing pills for 4 weeks, and angiotensin-converting enzyme inhibitors, selective angiotensin receptor antagonist, and beta-blockers for 2 weeks; hypertension could still be treated with calcium channel inhibitors and alpha-2 agonists). All included patients underwent an abdominal CT scan and had a successful AVS for subtype diagnosis. In our center, the AVS procedures were always performed without ACTH stimulation, and we used cortisol selectivity index (SI) and aldosterone/cortisol lateralization index (LI) cutoffs of ≥2 and ≥3, respectively. In the centers participating in the validation study, AVS procedures were usually performed under ACTH stimulation, and SI and LI cutoffs were >5 and >4, respectively. In patients with lateralized AVS, the diagnosis of UPA was confirmed by clinical and biochemical response evaluated at 6 months after adrenalectomy according to PASO criteria (primary aldosteronism surgical outcomes) (10). Only one patient with severe PA but negative imaging was first diagnosed with unilateral disease based on AVS results but showed no clinical or biochemical improvement post-adrenalectomy. This patient was considered to have inaccurate AVS and was included in the BPA group.

A multicenter cohort of 130 patients (67 patients with BPA and 63 patients with UPA based on AVS results) served as external validation. These patients were recruited from the Croatian University Hospital Center of Zagreb and from three Belgian academic hospitals (Hôpital Universitaire de Bruxelles (HUB) – Hôpital Erasme, Centre Hospitalier Universitaire de Liège, and Universitair Ziekenhuis Antwerpen).

The study was approved by the central Ethics Committee of the Cliniques Universitaires St-Luc (reference CEHF 2021/10MAR/119). In agreement with this Ethics Committee, patient informed consent was waived due to the retrospective design of the study. We also handled study data according to national laws and European General Data Protection Regulations.

### Hormonal assays

In the development cohort, PAC and direct renin concentration (DRC) were measured using automated immunoassays with chemiluminescence detection (analyzer: Liaison XL from Diasorin, Belgium). Before 2008, plasma renin activity was measured using an in-house enzymatic method. Plasma cortisol was determined using an automated competitive immunoassay with electrochemiluminescence detection (analyzer: Cobas 8000 e602 module from Roche, Belgium).

In the validation cohort, PAC was measured using automated chemiluminescence immunoassays (IDS ISYS, Immunodiagnostic Systems Limited, Boldon, UK in Zagreb; Liaison XL from Diasorin in Antwerpen and Brussels) or by liquid chromatography-mass spectrometry (LC-MS) (QTRAP 5500 LC-MS/MS System, Sciex, USA in Liège). Renin was measured either as plasma renin activity by radioimmunoassay (Angiotensine I RIA, DiaSource Immunoassay SA, Louvain-la-Neuve, Belgium in Zagreb, Liège, and in Brussels before 2011) or as direct renin by automated immunoassays with chemiluminescence detection (Liaison XL from Diasorin in Antwerpen, in Liège, and in Brussels after 2011). Depending on the aldosterone assays used in the different centers, the within- and between-assay coefficients of variation were between 3 and 6% and between 5 and 8%, respectively, for CLIA, and between 2.8 and 5.1% for LC-MS.

### Statistical analysis

Statistical analyses were conducted using IBM SPSS version 27. Continuous variables were reported as means ± standard deviations or medians with confidence intervals (P5-P95), depending on the distribution. Discrete variables were reported as frequencies. Continuous variables were compared using the student’s *t*-test or the Mann–Whitney U test, based on their distribution. Predictive factors for diagnosing UPA were identified using univariate and multivariate logistic regression. Univariate analysis was first conducted to find variables significantly associated with the diagnosis of UPA (*P* < 0.05). These variables were integrated into the KASAI score, provided they were not highly correlated. In such cases, the most significant variable was selected. A stepwise entry procedure was also used to determine whether some of these parameters remained independently significant. Receiver operating characteristic (ROC) curves were constructed for each parameter included in the score to determine the sensitivity and specificity of each value for diagnosing UPA. Depending on the results, 0, 1, or 3 points were respectively assigned to defined cutoff values (i.e., a score of 3 was given when specificity was above 60% for hypokalemia and 80% for supine aldosteronemia and post-SIT aldosteronemia). The diagnostic performance of the KASAI score was first established in 52 patients of the development cohort (missing data for SIT in 20 patients) and then confirmed in the separate multicenter validation cohort of 130 patients using ROC curves.

## Results

### Patient characteristics

The characteristics of the 72 patients included in the development cohort (51 with UPA and 21 with BPA) are shown in Table 1. The mean age at diagnosis was 52 years with a male-to-female ratio of 1.3. There was no significant clinical difference between the UPA and BPA subgroups, including the characteristics of hypertension and the prevalence of target organ damage. Hypokalemia was significantly more frequent, and its nadir was more severe in the UPA group than in the BPA group (78 vs 52% and 2.66 mmol/L vs 2.99 mmol/L, respectively, *P* < 0.05). Patients with unilateral disease also had significantly higher mean PAC values in supine position and after SIT. Adrenal nodules were detected by abdominal CT scan in 49/51 UPA patients (unilateral in 41 and bilateral in 8 patients). Adrenal nodules or hyperplasia were also detected in 14/21 BPA patients (unilateral abnormality in eight and bilateral in six patients). By comparing the imaging results with the results of the AVS, the CT alone could have been reliable for subtype diagnosis in only 54/72 (75%) patients. More precisely, among the 52 patients who demonstrated a lateralization gradient on AVS, 40 showed a concordant imaging result, while 12 patients had discordant findings: 10 had thin adrenal glands or bilateral symmetrical nodules, while 2 patients presented with a nodule located on the opposite side compared to the lateralization gradient. Notably, these 2 patients underwent adrenalectomy on the side indicated by the lateralization gradient and subsequently experienced favorable clinical and biochemical outcomes.

**Table 1:** Clinical and biochemical characteristics of the development cohort.

Variable	Total	UPA	BPA	*P* value
	*n* = 72	*n* = 51	*n* = 21	
Sex M/F (*n*, %)	41/31, 56.9%	29/22, 56.9%	12/9, 57.1%	0.983
Age at diagnosis (years)	52 ± 12	53 ± 13	51 ± 12	0.656
BMI (kg/m^2^)	28.3 ± 4.5	28.5 ± 4.7	27.6 ± 4.2	0.461
Diabetes (*n*, %)	20/72, 28%	11/51, 21.6%	9/21, 42.9%	0.067
Duration of hypertension (years)	12 (1–30)	10 (1–28)	12 (1–38)	0.093
Systolic blood pressure (mmHg)	154.1 ± 21.0	155.3 ± 19.0	151.1 ± 25.3	0.434
Diastolic blood pressure (mmHg)	91.9 ± 14.1	92.7 ± 14.6	90 ± 12.8	0.465
Antihypertensive drugs per patient at baseline (DDD)	3.8 ± 1.9	3.7 ± 2.2	3.9 ± 1.5	0.687
eGFR CKD-EPI (ml/min)	85.5 ± 25.8	85.7 ± 24.1	78.1 ± 29.4	0.261
Left ventricular hypertrophy (*n*, %)	15/68, 22.1%	9/47, 19.1%	6/21, 28.6%	0.370
Plasma aldosterone at screening (ng/dL)	31.4 (17.8–87.3)	33.0 (17.4–93.9)	24.9 (17.4–85.3)	0.304
Direct renin concentration at screening (μUI/mL)	1.7 (0.5–8.5)	1.7 (0.5–9.6)	2.6 (0.5–6.4)	0.846
ARR at screening (ng/dL/μUI/mL)	16.4 (4.6–132.0)	16.4 (4.7–116.4)	15.4 (4.4–104.6)	0.958
Supine plasma aldosterone (ng/dL)	28.50 (8.91–93.5)	35.7 (13.01–101.03)	21.62 (8.91–57.34)	0.013
Lowest potassium level (mmol/L)	2.77 ± 0.52	2.66 ± 0.46	2.99 ± 0.51	0.019
Potassium <3.5 mmol/L (*n*, %)	51/72, 70.8%	40/51, 78.4%	11/21, 52.4%	0.027
Plasma aldosterone after saline infusion test (ng/dL)	19.4 (8.88–66.38)	25.7 (11.17–78.70)	14.41 (6.12–36.89)	0.023

M, men; F, female; BMI, body mass index; DDD, defined daily dose; eGFR, estimated glomerular filtration rate; ARR, renin to aldosterone ratio.

### Development of the KASAI score

Univariate logistic regression analysis showed that nadir serum potassium value, supine PAC, PAC at time 4 h of SIT, and positive adrenal imaging results (unilateral nodule) were significantly associated with a diagnosis of UPA, while other parameters such as age, sex, PAC measured in a sitting position, or aldosterone-to-renin ratio were not (Table 2). When the four most significant parameters were introduced in the multivariate analysis (using kalemia <3 mmol/L as the best predictor in univariate analysis), none was independently associated with the presence of UPA (except for a tendency for PAC after SIT), thus suggesting valuable contribution of each variable in the predictive model.

**Table 2:** Uni- and multivariate logistic regression analyses performed on several parameters potentially used for subtyping primary aldosteronism in the development cohort (*n* = 52 patients).

Variable	Univariate analysis			Multivariate analysis		
	OR	95% CI	*P* value	OR	95% CI	*P* value
Age at diagnosis (years)	0.651	0.949–1.033	0.561	-	-	-
Male sex	1.011	0.362–2.829	0.983	-	-	-
Lowest potassium level (mmol/L)	0.213	0.063–0.721	0.013	-	-	-
Kalemia ≤3.5 mmol/L	3.727	1.240–11.204	0.019	-	-	-
Kalemia ≤3.0 mmol/L	4.848	1.627–14.448	0.005	3.041	0.658–14.063	0.155
ARR at screening (ng/dL/μUI/mL)	1.000	0.985–1.015	0.957	-	-	-
Supine aldosterone (ng/dL)	1.044	1.007–1.082	0.018	1.005	0.958–1.055	0.840
Plasma aldosterone after SIT (ng/dL)	1.068	1.005–1.136	0.034	1.064	0.985–1.150	0.115
% decrease in aldosterone during SIT	1.026	1.001–1.052	0.039	-	-	-
Unilateral nodule at imaging	4.294	1.467–12.573	0.008	2.672	0.642–11.122	0.177

ARR, renin to aldosterone ratio; SIT, saline infusion test; OR, odds ratio; CI, confidence interval. Due to the high correlation between these parameters, kalemia ≤3.0 mmol/L was chosen rather than kalemia ≤3.5 mmol/L in multivariate analysis, and absolute plasma aldosterone concentration after SIT was chosen rather than the %-decrease in aldosterone during SIT because of higher significance in univariate analyses.

Based on the ROC curves generated for each laboratory parameter, we selected the cutoffs to assign 0, 1, or 3 points: minimal serum potassium (0: >3.4 mmol/L; 1: between 3.0 and 3.4 mmol/L; and 3: <3.0 mmol/L), supine PAC (0: <25 ng/dL; 1: 25 to 45 ng/dL; 3: > 45 ng/dL), PAC after SIT (0: <20 ng/dL; 1: 20 to 25 ng/dL; 3: > 25 ng/dL). For the adrenal imaging results, we distinguished between no or symmetric bilateral abnormality (0 point), asymmetric bilateral abnormality (bilateral hyperplasia or nodule with a size difference greater than 1 cm – 1 point), and unilateral nodule (3 points). The sum of the points assigned to each parameter corresponded to the KASAI score (maximal score of 12) assigned to each patient (Table 3).

**Table 3:** Definition of the KASAI score.

	Variable	0 point	1 point	3 points
K	Lowest potassium level (mmol/L)	>3.4	3–3.4	<3
A	Supine aldosterone (ng/dL)	<25	25–45	>45
SA	Plasma aldosterone after saline infusion test (ng/dL)	<20	20–25	>25
I	Adrenal imaging	No lesion, bilateral hyperplasia, bilateral nodule (size difference <1 cm)	Asymmetric bilateral hyperplasia or nodule (size difference >1 cm)	Unilateral nodule or hyperplasia

**Figure 1:**
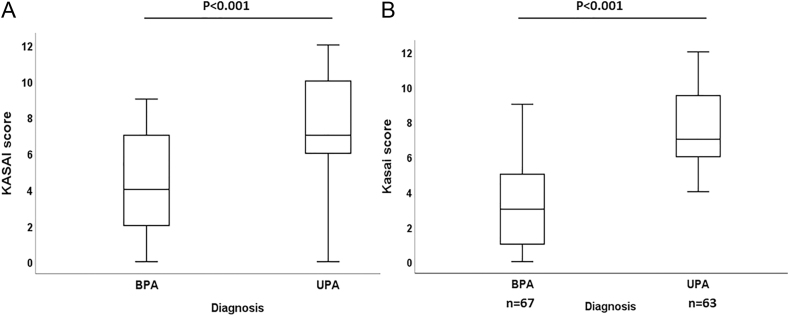
The KASAI score calculated in the development cohort (panel A) and in the validation cohort (panel B). UPA: unilateral primary aldosteronism; BPA: bilateral primary aldosteronism.

**Figure 2:**
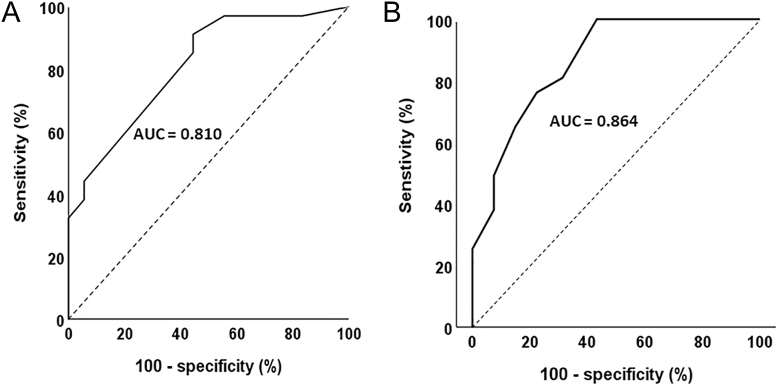
ROC curve for discrimination of UPA using the KASAI score in the development (panel A) and validation cohorts (panel B).

**Figure 3:**
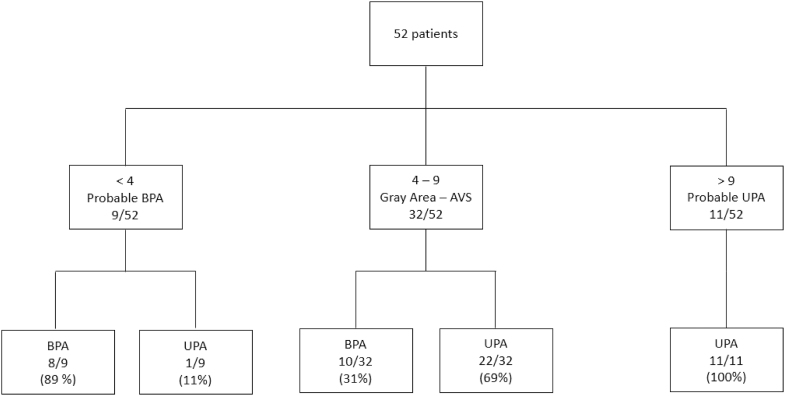
Proportions of primary aldosteronism subtypes (BPA: bilateral primary aldosteronism, UPA: unilateral primary aldosteronism, and AVS: adrenal venous sampling) depending on KASAI score in the primary cohort.

The KASAI score could be calculated for 52 patients in our cohort (SIT not performed in 20 patients). As expected, the UPA group had a significantly higher KASAI score than the BPA group (*P* < 0.001) (Fig. 1A). The area under the ROC curve for discrimination of UPA from BPA was 0.81 (95% CI, 0.70–0.90) in our primary cohort (Fig. 2A). A score greater than 9/12 had a 100% specificity to identify a UPA, a score less than 4/12 had 89% specificity to diagnose BPA, while performing AVS remained indicated for a score between 4 and 9 (gray area). The score may have avoided performing AVS in 40% of the patients, correctly identifying 11 UPA patients with a score >9 and 8 BPA patients with a score <4 (Fig. 3). Only one UPA patient with a score of 3 would have been falsely identified as BPA and treated medically, while no patient would have undergone unnecessary surgery.

Interestingly, among the 11 patients with a KASAI score greater than 9; all demonstrated concordant results between lateralization gradients and imaging findings (unilateral nodule).

### Validation cohort

**Figure 4:**
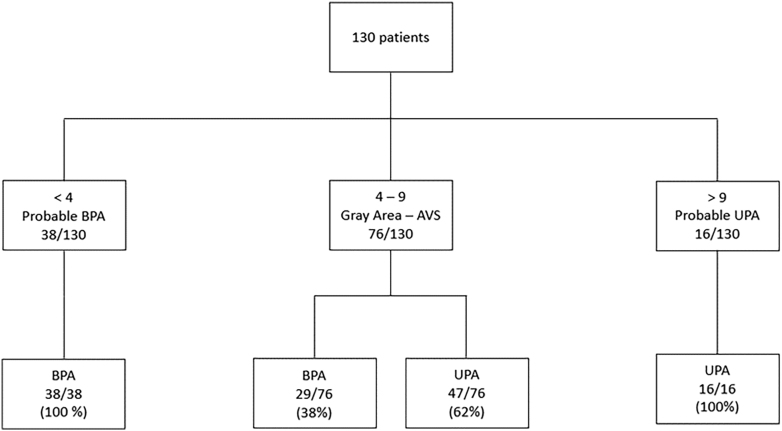
Proportions of primary aldosteronism subtypes (BPA: bilateral primary aldosteronism, UPA: unilateral primary aldosteronism, and AVS: adrenal venous sampling) depending on KASAI score in the validation cohort.

The characteristics of the 130 patients included in the validation cohort are shown in Supplemental Table 1 (see section on Supplementary materials given at the end of the article). This multicenter cohort included 63 subjects with UPA and 67 with BPA. The characteristics of these patients were very similar to our development cohort, with a mean age of 52 years and a male-to-female ratio of 1.50. The KASAI score was also significantly higher in the UPA than in the BPA subgroup (Fig. 1B, *P* < 0.001), and the area under the ROC curve (AUC) using the score for discrimination of UPA from BPA was 0.86 (95% CI, 0.80–0.90) (Fig. 2B), slightly better than in the derivation cohort. The score may have avoided the AVS in 54/130 cases (42%), correctly identifying with a 100% specificity 38 UPA patients (score >9) and 16 BPA patients (score <4) (Fig. 4). Of note, after excluding five patients from one center with aldosterone measured by LC-MS, the ROC curve established for the 125 remaining patients was identical (AUC 0.86), as were the sensitivities and specificities of the KASAI score (data not shown).

### Comparison with the SPACE score

To evaluate the diagnostic performance of the KASAI score further, we compared our score with the validated SPACE score (9, 11). The distribution of our development and validation cohorts according to SPACE score is illustrated in Supplemental Figs 1 and 2. Based on accepted cutoffs of <8.5 and >16 to diagnose BPA and UPA, respectively, the SPACE score bypassed the need for AVS in 63% of patients in the development cohort and 62% in the validation cohort. However, 1/52 and 8/63 UPA patients would have been misclassified as BPA in the primary and validation cohorts, respectively, while 6/21 and 10/67 BPA patients would have been misclassified as UPA in the primary and validation cohorts, respectively. Thus, in comparison with the SPACE score, the diagnostic performance of the KASAI score was much better in terms of specificity and positive predictive value to diagnose UPA, while the negative predictive values were similar (Table 4).

**Table 4:** Comparison between a KASAI score >9 and a SPACE score >16 to predict UPA in the development and validation cohorts.

	Development cohort (*n* = 52; 34 UPA – 18 BPA)				Validation cohort (*n* = 130; 63 UPA – 67 BPA)			
	Sensitivity (%)	Specificity (%)	PPV (%)	NPV (%)	Sensitivity (%)	Specificity (%)	PPV (%)	NPV (%)
KASAI score >9	11/34 (29%)	18/18 (100%)	11/11 (100%)	18/41 (44%)	16/63 (25%)	67/67 (100%)	16/16 (100%)	67/114 (59%)
SPACE score >16	20/34 (59%)	12/18 (66%)	20/26 (77%)	12/26 (46%)	33/63 (52%)	57/67 (85%)	33/43 (77%)	57/87 (66%)

UPA, unilateral primary aldosteronism; BPA, bilateral primary aldosteronism; PPV, positive predictive value; NPV, negative predictive value.

## Discussion

Given the high prevalence of PA and the limited availability of AVS to differentiate between UPA and BPA, there is a need to develop alternatives to this invasive procedure. We propose a new noninvasive and simple predictive diagnostic score (KASAI score) that can reduce the number of required AVS while retaining a high diagnostic specificity and predictive value to recognize surgically remediable forms of PA. Using our proposed cutoffs, the KASAI score may have avoided the need for AVS in 40% of the patients with minimal misclassification. This implies a reduction in medical costs and faster management in a subset of patients. It also reallocates AVS resources to patients with a score within the gray area. Moreover, the incorporated variables in the KASAI score are readily available from the diagnostic procedure leading to confirmation of PA.

The KASAI score has a high probability of identifying UPA as it is targeting the most severe forms of PA (12). Hypokalemia is found in only 9–37% of all cases of PA but is much more prominent in patients with an aldosterone-producing adenoma (13, 14). In a large prospective study of the prevalence of PA, spontaneous hypokalemia <3.5 mmol/L was found in only 17% of patients with bilateral PA compared to about 50% of patients with unilateral disease (15). Hypokalemia is used as a criterion in almost every scoring system to predict UPA, but with different cutoffs values (8). Using a lower serum potassium cutoff of 3.0 mmol/L greatly improves the positive predictive value when diagnosing UPA.

A high PAC in resting conditions is also a reflection of severe PA. Supine morning PAC is a more reliable measurement, as variations of PAC are expected in response to the diurnal rhythm and activity (16), and this was the case in our study as ambulatory aldosterone measured in a sitting position was not a significant predictor of UPA. One study already showed that a supine PAC value above 32 ng/dL could be used as a good indicator for the subtype diagnosis (17).

The SIT is the most widely used diagnostic confirmatory test for PA (18). Recent studies have shown that patients with UPA display a smaller aldosterone reduction after SIT compared to patients with bilateral PA (19, 20). We believe that considering the absolute PAC value after saline suppression further increases the accuracy of the prediction model. It must, however, be noted that concerns have been raised about the validity of some plasma aldosterone immunoassays, which can return inaccurate results (21), and therefore well-validated assays must be used in order to use predictive scores efficiently. Nevertheless, it is interesting to note that our predictive score worked as well in a validation cohort of patients in whom several aldosterone assay platforms were used.

Adrenal imaging results are not considered a reliable indicator of the lateralization of PA (5, 22, 23). Abdominal CT scan has a sensitivity and a specificity of 78 and 75%, respectively, which are well below the performance of the AVS (respectively 95 and 100%), even for patients less than 35 years old (24). A study carried out on a cohort of 1,625 patients who underwent AVS showed that imaging failed to identify lateralization of aldosterone production in 28% of patients (22). Nevertheless, one should keep in mind that CT (or MR) imaging is a necessary step in order to localize and characterize the diseased adrenal gland before surgery, in particular in the absence of AVS. Our score should be used more carefully in older patients, as the frequency of adrenal incidentalomas is more important in this category of the population (25). However, due to a limited contribution of imaging to the KASAI score, the presence of an adrenal nodule without concomitant indices of severe PA will never classify an individual as having UPA. The development of functional imaging could provide a more reliable method for localizing the hyperfunctional gland(s), alongside the use of a severity score to confirm the unilateral nature of PA (7).

On the other hand, Kocjan *et al.* have developed prediction criteria able to identify less severe forms of hyperaldosteronism, characterized by normokalemia (>3.5 mmol/L) and post-SIT aldosterone <18 ng/dL (26). When combined with symmetric adrenal imaging (absence of abnormalities or bilateral nodules), these parameters are consistently predictive of BPA with a specificity of 100% in their experience. The KASAI score also demonstrated a high specificity to identify BPA when the score is below 4, as only one of these patients had a final diagnosis of UPA. Another score proposed by Kamemura *et al.*, comprising CT findings and 1 point each for kalemia >3.8 mEq/L, aldosterone-to-renin ratio ≤550 pg/mL/ng/mL/h, and female sex (27), had the highest specificity to predict BPA in a recent meta-analysis (95% with a sensitivity of 28%) (8). In our study, using the sex ratio was not discriminative. This discrepancy could be explained by the high prevalence of adenoma related to KCNJ5 mutation in Japan, a mutation that is more frequent in women than in men (28). As these adenomas are associated with a production of 18-hydroxy and 18-oxocortisol metabolites, it is possible that measuring these products could help in identifying UPA (12).

Nevertheless, none of the previously mentioned algorithms have been tested in other independent cohorts, which typically lead to lower diagnostic performance. Validation of the KASAI score in a multicenter independent cohort confirms its diagnostic performance. When comparing our score with the latest described score-based algorithm, the SPACE score, proposed by Burrello *et al.* (11), we could demonstrate a slight superiority, at least regarding specificity and positive predictive value of a high score. The SPACE score stands out for having been tested both on an internal validation cohort of 65 patients and an external validation cohort of 118 patients. It was also evaluated later by Kocjan *et al.* (9). The accuracy of this score, using a cutoff of 16/20 for diagnosing UPA, decreased from 81% in the internal validation cohort to 79% in the external validation cohort, and further to 67% in Kocjan’s cohort. Although the parameters identified by logistic regression in our cohort were similar to those used in the SPACE score, the latter places significant emphasis on imaging results (12.5/20 points) and potassium levels (5/20 points). Consequently, the score for a unilateral adrenal nodule (10.5/20 points) larger than 1 cm (1/20 points), associated with a potassium level below 3.4 mmol/L (5/20 points), is sufficient to recommend adrenalectomy, regardless of the aldosterone levels measured in supine position and at the end of the confirmation test. However, as mentioned before, adrenal incidentalomas are common in elderly patients (25), who are also at risk of hypertension and mild hypokalemia. Thus, in our own cohort of 67 patients diagnosed with BPA, nine would have been mistakenly operated on based on the SPACE score. Because of this lack of specificity, the Endocrine Society PA guidelines (5) recommend performing an adrenalectomy without AVS in patients with isolated adrenal nodule and hypokalemia only in patients under 35 years and aldosterone levels above 30 ng/dL. The KASAI score spared fewer patients from AVS compared to the SPACE score (41 vs 63%) but did so more judiciously.

The strength of our score lies in its easy applicability and alignment with other scores in terms of diagnostic criteria, while modifying the cutoff values to enhance precision. The score reliably diagnoses both BPA and UPA at both ends of the spectrum. The external validation showed similar performance.

The main limitation of this study is the retrospective inclusion of patients. The lower percentage of patients with bilateral PA included in the development cohort might have led to a selection bias. However, it showed similar performance in the external cohort containing a greater number of BPA patients. Due to the retrospective, multicenter nature of the study spanning several decades, no standardized AVS protocol was applied. Importantly, since the KASAI score does not incorporate AVS-derived parameters, variability in AVS methodology would primarily affect the final diagnosis rather than the score itself. The KASAI score has not yet been validated in cohorts where AVS was either not performed or yielded noninterpretable results. Moreover, the score was developed using SIT conducted in the supine position, a protocol that typically requires a 24 h hospitalization. This requirement may limit its feasibility, particularly in resource-constrained settings. Nevertheless, the score could potentially reduce the need for AVS in a substantial subset of patients. Further validation in cohorts undergoing SIT in the seated position would be valuable. The KASAI score cannot be applied to patients with PA diagnoses by furosemide or captopril testing. In addition, low-dose dexamethasone suppression testing was not systematically performed across the cohort. This may represent a limitation, as autonomous cortisol secretion can influence aldosterone levels and confound the interpretation of diagnostic tests for PA. Finally, variations in screening test cutoff values and assay techniques across centers could affect generalizability (18). Our KASAI score has been well validated using aldosterone measurements by CLIA methodology, but it should now be validated in a larger cohort of patients with aldosterone measured by LC-MS. Further studies performed in other expert centers are therefore welcome to confirm the validity of this new KASAI score.

In conclusion, we propose a new biochemical-radiological score that could simplify the subtyping diagnostic algorithm of PA. The KASAI score accurately predicts UPA when above 9/12 and BPA when below 4/12, allowing bypass of AVS in 40% of the patients with confirmed primary aldosteronism.

## Supplementary materials



## Declaration of interest

The authors declare that there is no conflict of interest that could be perceived as prejudicing the impartiality of the work reported.

## Funding

This work did not receive any specific grant from any funding agency in the public, commercial, or not-for-profit sector.

## Ethics

The study was approved by the central Ethics Committee of the Cliniques Universitaires St-Luc (reference CEHF 2021/10MAR/119). In agreement with this Ethics Committee, patients’ informed consent was waived due to the retrospective design of the study. We also handled study data according to national laws and European General Data Protection Regulations.
