# Supplementary material for: Validation of a new non-invasive predictive score (KASAI) for primary aldosteronism subtyping
Source: Endocr Connect. 2025 Sep 3;14(9):e250156. doi: 10.1530/EC-25-0156 (PMC12412202; doi:10.1530/EC-25-0156)

Supplemental Table: Clinical and biochemical characteristics of the validation cohort

Variable	Total N=130	UPA N=63	BPA N=67	P value
Sex M/W (n, %)	78/52, 60.0%	35/28, 55.6%	43/24, 64.1%	0.205
Age at diagnosis (years)	52 ± 11	50 ± 10	53 ± 13	0.135
BMI (kg/m^2)	29.8 ± 5.8	29.0 ± 5.9	30.6 ± 5.7	0.121
Systolic Blood Pressure (mmHg)	149.5 ± 20.6	150.2 ± 20.4	148.8 ± 21.0	0.692
Diastolic Blood Pressure (mmHg)	92.6 ± 14.9	92.8 ± 14.7	92.4 ± 14.8	0.985
Antihypertensive drugs per patient at baseline (DDD)	4.6 ± 3.1	5.5 ± 3.5	3.8 ± 2.3	0.003
eGFR CKD-EPI (ml/min)	92.3 ± 22.2	94.4 ± 24.7	90.3 ± 19.6	0.291
Aldosterone at screening (ng/dL)	28.7 [15.1-85.3]	38.1 [16.9-94.4]	21.9 [14.1-77.5]	<0.001
Lowest potassium level (mmol/L)	3.20 ± 0.64	2.91 ± 0.56	3.47 ± 0.59	<0.001
Aldosterone after saline infusion test (ng/dL)	19.5 [10.6-77.2]	24.7 [12.8-98.1]	14.5 [10.2-46.9]	0.001

M: Men, F: Female, BMI: Body Mass Index, DDD: Defined daily dose; eGFR: Estimated Glomerular Filtration Rate

Supplemental Figure 1. Proportion of primary aldosteronism subtypes (BPA: Bilateral Primary Aldosteronism, UPA: Unilateral Primary Aldosteronism, AVS: Adrenal Venous Sampling) depending on SPACE score in the development cohort

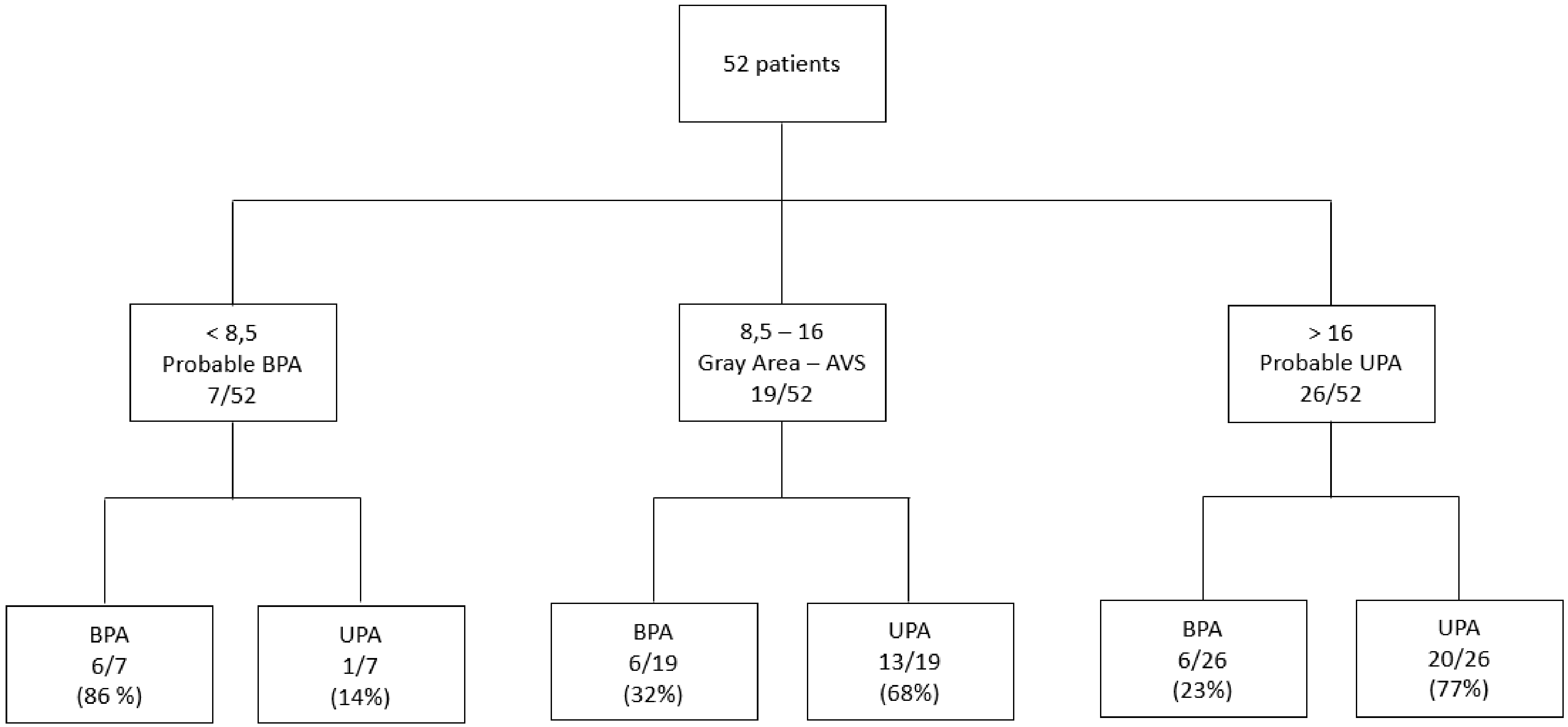

Supplemental Figure 2. Proportion of primary aldosteronism subtypes (BPA: Bilateral Primary Aldosteronism, UPA: Unilateral Primary Aldosteronism, AVS: Adrenal Venous Sampling) depending on SPACE score in the validation cohort

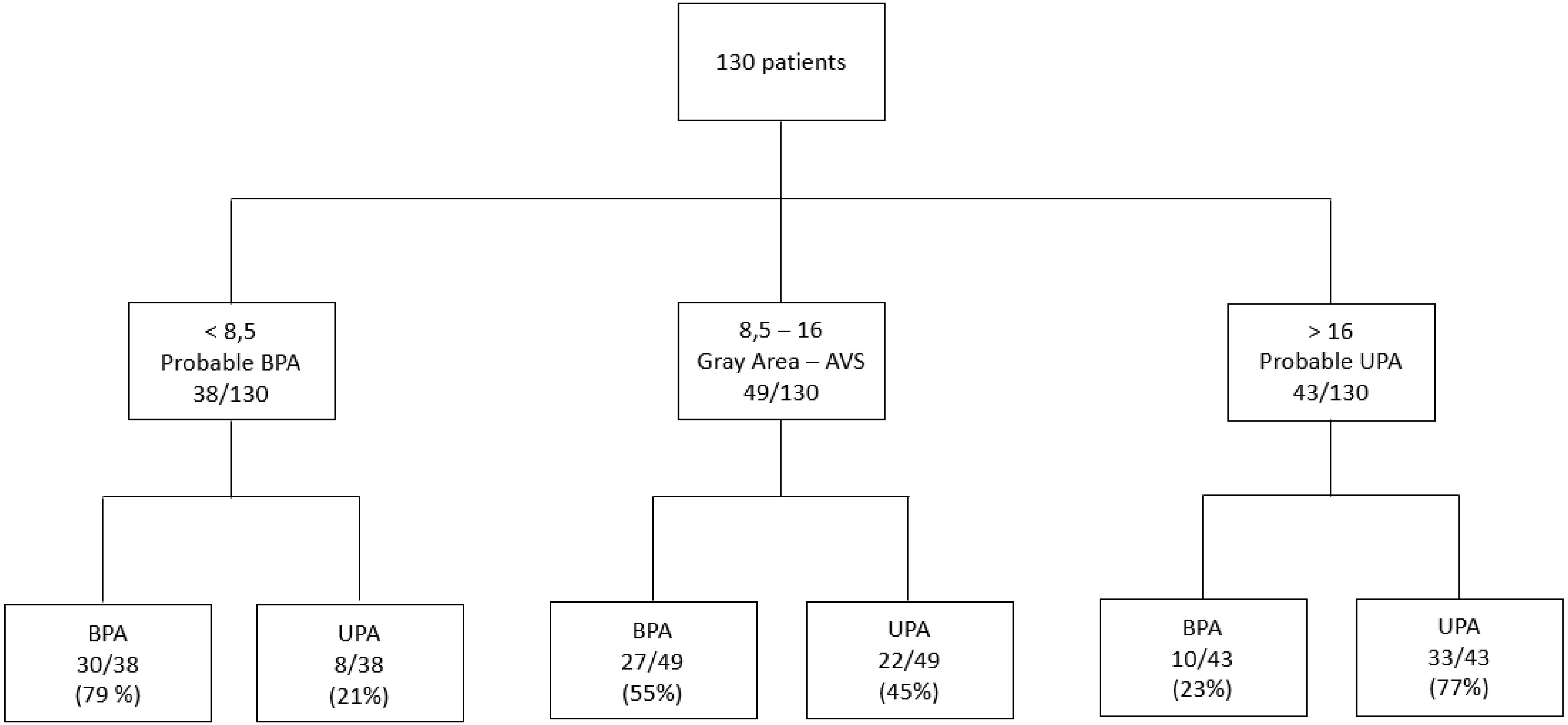

Supplement: Supplementary file 1 [file supplementary_materials.pdf]
